# Dynamic contrast-enhanced ultrasound perfusion analysis for preoperative prediction of aggressive hepatocellular carcinoma subtypes

**DOI:** 10.1186/s13244-025-02052-z

**Published:** 2025-09-23

**Authors:** Xin Guan, Xiao Li, Daohui Yang, Chongke Zhao, Dan Lu, Yaqin Zhang, Yikang Sun, Boyang Zhou, Zitong Chen, Xinyuan Hu, Hong Han, Qing Lu, Huixiong Xu, Lifan Wang

**Affiliations:** 1 Department of Ultrasound, Zhongshan Hospital (Xiamen), Fudan University, Xiamen, China; 2 Department of Ultrasound, Institute of Ultrasound in Medicine and Engineering, Zhongshan Hospital, Fudan University, Shanghai, China; 3 Shanghai Institute of Medical Imaging, Fudan University, Shanghai, China; 4 Department of Medical Ultrasound, Shanghai Tenth People’s Hospital, Shanghai Engineering Research Center of Ultrasound Diagnosis and Treatment, School of Medicine, Tongji University, Shanghai, China

**Keywords:** Contrast-enhanced ultrasound, Quantitative ultrasound, Hepatocellular carcinoma, Macrotrabecular-massive subtype

## Abstract

**Objective:**

To evaluate the potential of dynamic contrast-enhanced ultrasound (CEUS) quantitative parameters in preoperative prediction of macrotrabecular-massive (MTM) subtype and high Ki-67 pattern in hepatocellular carcinoma (HCC) patients.

**Materials and methods:**

This study included a retrospective primary cohort and a multicenter prospective validation cohort comprising HCC patients who underwent surgical resection and preoperative CEUS between January 2023 and April 2024. The Clinic-CEUS model was established by combining clinical data and CEUS features, while the Clinic-Q-CEUS model was constructed by combining clinical data and CEUS features with matched quantitative parameters. Model performance was tested with the area under the receiver operating characteristic curve (AUC) in the validation cohort.

**Results:**

A total of 170 patients (mean age, 61 years ± 11 [SD]; 130 men; primary cohort, *n* = 118; validation cohort, *n* = 52) were included. The Clinic-Q-CEUS model better predicted MTM subtype and high Ki-67 pattern than the Clinic-CEUS model (AUC, 0.860 vs. 0.753, *p* = 0.027 and AUC, 0.836 vs. 0.738, *p* = 0.036) in the primary cohort, with similar performance in the validation cohort (AUC, 0.868 vs. 0.693, *p* = 0.046 and AUC, 0.787 vs. 0.610, *p* = 0.018).

**Conclusions:**

Dynamic CEUS quantification analysis could be used as an effective adjunct tool for preoperative identification of MTM subtype and high Ki-67 pattern in HCC patients.

**Critical relevance statement:**

Dynamic contrast-enhanced ultrasound (CEUS) quantitative parameters can help radiologists more accurately identify aggressive macrotrabecular-massive (MTM) subtype and high Ki-67 pattern in HCC patients preoperatively, which provides useful information for subsequent treatment planning.

**Key Points:**

- Macrotrabecular-massive (MTM) subtype and high Ki-67 pattern in HCC affect prognosis, but diagnosis relies on invasive histopathology.
- A clinical-Q-CEUS model performed well in preoperative predicting aggressive HCC subtypes.
- Quantitative parameters of dynamic CEUS can provide valuable information to help accurately identify aggressive HCC subtypes.

**Graphical Abstract:**

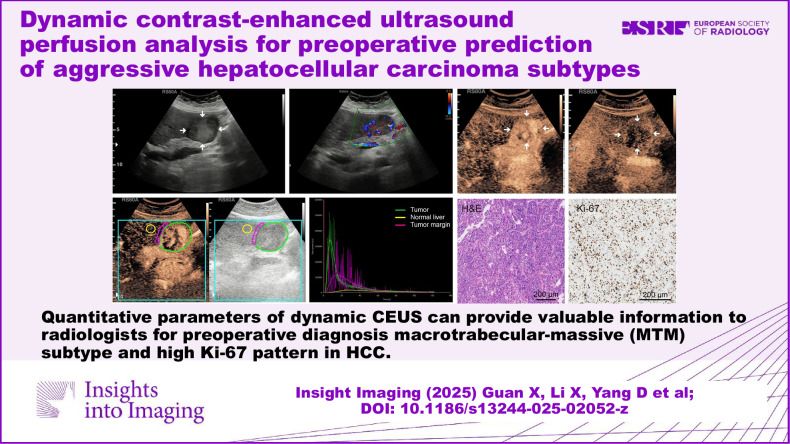

## Introduction

Primary liver cancer ranks as the sixth most common cancer and the third leading cause of cancer-related deaths worldwide, with hepatocellular carcinoma (HCC) accounting for more than 80% of cases [1]. The overall prognosis for patients with HCC is unsatisfactory and varies by subtype [2]. Several aggressive HCC subtypes with correlations to clinical outcomes have been identified. The macrotrabecular-massive (MTM) subtype is defined by the existence of > 50% macrotrabecular architecture (> 6 cells thick), while Ki-67 is a biomarker of cell proliferation that reflects tumor proliferative activity [3]. Both MTM subtype and high Ki-67 pattern are considered to be related to postoperative recurrence and poor clinical outcome [3–5]. However, they can only be confirmed by invasive histopathology. Preoperative identification of aggressive MTM subtype or high Ki-67 pattern may contribute to personalized treatment planning and improved prognosis [3, 6].

Previous studies have illustrated that several noninvasive imaging features from contrast-enhanced computed tomography and contrast-enhanced magnetic resonance imaging correlate with aggressive HCC subtypes [7–10]. Specifically, contrast-enhanced ultrasound (CEUS) has been widely applied in the diagnosis, surveillance and follow-up of HCC [11]. It leverages vascular perfusion information to analyze lesion nature and thus offers real-time, noninvasive visualization of HCC. However, limited studies have demonstrated that CEUS features could be used to identify patients with suspected aggressive HCC subtypes [12, 13]. Moreover, it is important to recognize that CEUS examination relies heavily on operator experience, which makes interpretation of the results susceptible to subjective interference. Thus, objective assessment of CEUS video with the help of quantitative tools may help improve the diagnostic accuracy for aggressive HCC subtypes.

Dynamic CEUS permits quantitative assessment of tumor perfusion via time intensity curves (TIC), resulting in a more objective quantitative analysis of subtle differences in enhancement degree and minimizing the influence of operator subjectivity [14, 15]. The application of Vuebox^®^ software for quantitative analysis of dynamic CEUS in tumors of breast, pancreas, thyroid and liver has been reported, but its use to identify MTM-HCC and Ki-67 pattern has not yet been seen [16]. The aim of this study was to evaluate the potential of dynamic CEUS quantification analysis using Vuebox^®^ software in preoperative distinguishing MTM subtype and high Ki-67 pattern in HCC patients, and to develop a prediction model based on clinical data, CEUS features and relative quantification parameters to identify MTM-HCC as well as high Ki-67 pattern.

## Materials and methods

### Patients

This study was approved by the Ethics Committee of Zhongshan Hospital of Fudan University (No: B2022-569R), and the requirement to obtain patient’s informed consent was waived for the retrospective cohort. Written informed consent was obtained from all participants in the prospective validation study. This study included a retrospective single-center primary cohort for identification of independent features, prediction model development and a prospective two-center validation cohort for prediction model testing in subtype identification.

For the primary cohort, consecutive HCC patients confirmed by pathology who underwent preoperative CEUS between January 2023 and December 2023 at the Zhongshan Hospital of Fudan University (Institute 1) were studied. Patients were enrolled if they met the following criteria: (1) Liver CEUS using Sonovue^®^ (Bracco) was performed within 1 month before surgery, (2) HCC diagnosis was reached by postoperative pathological examination, (3) not received any preoperative treatment. The exclusion criteria were as follows: (1) poor CEUS video or digital imaging and communications in medicine (DICOM) record less than 120 s for interpretation, (2) lack of required clinical or pathological information. HCC patients who satisfied the above criteria at the Zhongshan Hospital (Xiamen) of Fudan University (Institute 2) and the Institute 1 between January 2024 and April 2024 constituted the validation cohort.

### Histopathologic examination

Histopathologic evaluation was available after surgery for all HCC patients. Histopathologic features were recorded as follows: tumor size, lesion numbers, Edmonson-Steiner grade, cirrhosis, satellite nodules, macrovascular invasion, microvascular invasion and capsular invasion. Two abdominal pathologists who were unaware of the CEUS information reviewed all surgical slices. The largest diameter tumor was analyzed when multiple tumors were present in one patient. The MTM subtype is defined as a macrotrabecular (trabecular thickness of more than 6 cells) architectural pattern in over 50% tumor area at hematoxylin-eosin staining [3]. Tumor sections were stained with Ki-67 antibody, and high Ki-67 pattern is defined as > 20% immune-positive cells [13, 17].

### CEUS imaging analysis

To ensure the reliability of imaging interpretations, the US and CEUS images were reviewed independently by two radiologists (with more than 5 years of experience, respectively) who were unaware of patient information. When there is an inconsistency, a third radiologist with 20 years of abdominal CEUS experience is consulted. If the patient had multiple tumor lesions, the largest tumor lesion was evaluated. The senior radiologist re-evaluated all cases after a 4-week interval to minimize recall bias. The following characteristics were evaluated for each lesion: (1) tumor size, (2) location, (3) echogenicity, (4) shape, (5) margin, (6) blood flow signal, (7) halo sign, (8) intratumoral necrosis, (9) intratumoral artery, (10) peritumoral nutrient vessel, (11) arterial phase (AP) enhancement pattern, (12) AP enhancement capsule, (13) AP branched enhancement, (14) portal vein phase (PVP) enhancement degree, (15) late phase (LP) enhancement degree. The American College of Radiology CEUS Liver Imaging Reporting and Data System (LI-RADS) category (version 2017) was applied to all patients.

### Quantitative analysis of dynamic CEUS

The CEUS DICOM videos were offline analyzed using VueBox^®^ software (Bracco) by an independent radiologist with 5 years of experience, who was not informed of both clinicopathological and CEUS information. Prior to analysis, the DICOM video was calibrated using a specific calibration file matched to the ultrasound system. The whole contrast process was systematically observed and four regions of interest (ROI) were manually outlined. Border ROI (cyan) was a square area containing the lesion area and partially normal liver tissue. Lesion ROI (green) was drawn within border ROI based on tumor features. Reference ROI (yellow) was outlined within normal liver tissue near the tumor with a predetermined area size (about 1 cm^2^). The liver parenchyma around 3 mm outside the lesion was defined as margin ROI (magenta). The depths of the ROIs (except for the border ROI) were kept as similar as possible while ensuring TIC curve fit quality greater than 75%, and care was taken to avoid the surrounding major blood vessels. Motion compensation was adopted to minimize respiratory motion artifacts. The built-in algorithm then automatically generated TIC with various quantitative parameters including average contrast signal intensity (MeanLin), peak enhancement (PE), wash-in area under the curve (WiAUC), rise time (RT), mean transit time local (mTTl), time to peak (TTP), wash-in rate (WiR), wash-in perfusion index (WiPI), wash-out area under the curve (WoAUC), wash-in and wash-out area under the curve (WiWoAUC), fall time (FT) and wash-out rate (WoR). A TIC curve fit quality of more than 75% was considered successful for analysis.

### Statistical analysis

Continuous variables are displayed as means and standard deviations (SD), and categorical variables are displayed as numbers and percentages. Differences between different groups were analyzed by applying unpaired Student *t*-test or Mann–Whitney test for continuous variables and Fisher’s exact test or Chi-Squared test for categorical variables. Univariable and multivariable logistic regression analyses were used to identify independent predictors for aggressive HCC subtypes, which were combined for prediction model construction. The cutoff value of quantitative parameters was calculated using the Youden index, and the diagnostic performance was evaluated by receiver operating characteristic curve (ROC) analysis. Interobserver and intra-observer agreement for each US and CEUS feature was assessed by using the Cohen’s kappa statistic. The DeLong’s test was used to compare the AUC (95% CI) between the two models. The McNemar test was used to compare the sensitivity, specificity and accuracy of the two models. All statistical analyses were performed using SPSS software (version 27.0, IBM). A two-sided *p*-value < 0.05 indicated a statistically significant difference.

## Results

### Clinical and histopathologic characteristics

**Fig. 1:**
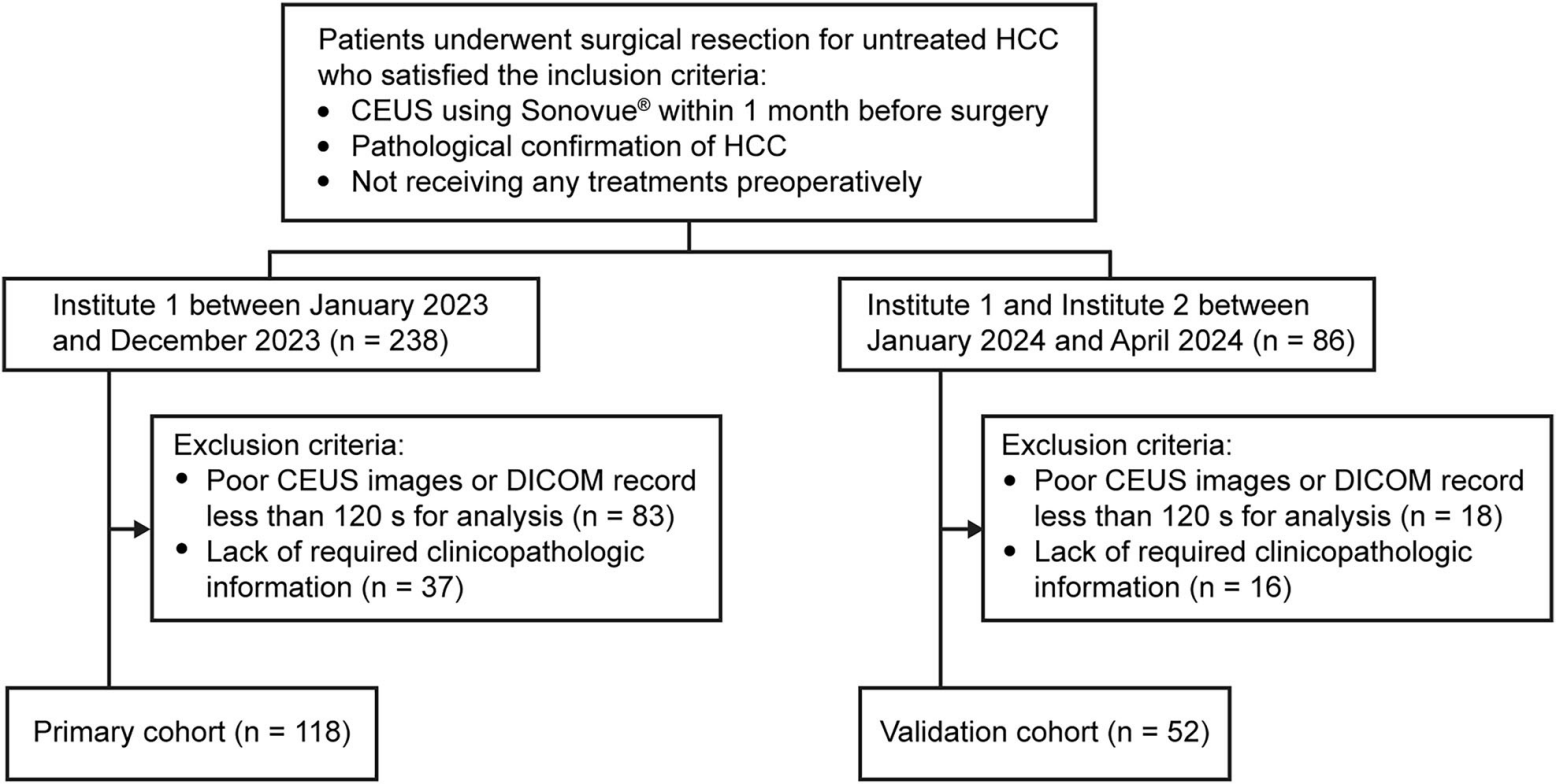
Flowchart of patient selection. HCC, hepatocellular carcinoma; CEUS, contrast-enhanced ultrasound; DICOM, digital imaging and communications in medicine

**Fig. 2:**
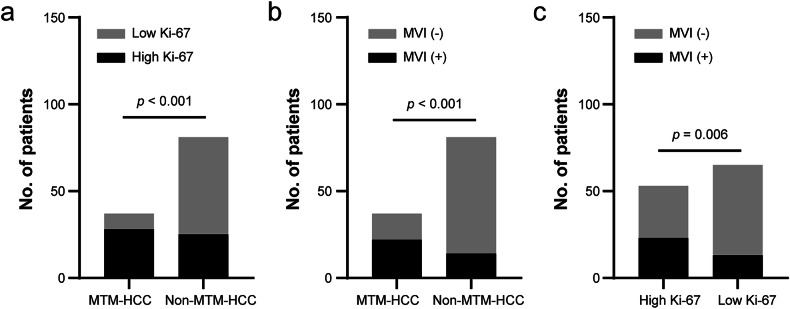
Bar charts exhibit the ratio association between MTM subtype and high Ki-67 pattern in HCC patients (**a**), between MTM subtype and MVI status (**b**) and between high Ki-67 pattern and MVI status (**c**). MTM, macrotrabecular-massive; HCC, hepatocellular carcinoma; MVI, microvascular invasion

Between January 2023 and December 2023, a total of 118 patients (mean age, 61 years ± 10 [SD]; 88 men) were enrolled in the primary cohort, and 52 patients (mean age, 62 years ± 11 [SD]; 42 men) were enrolled in the validation cohort (Fig. 1 and Table 1). The comparison of clinical and histopathologic characteristics displayed that patients with lens culinaris agglutinin-bound fraction of α-fetoprotein (AFP-L3%) > 10 U/mL were fewer in the primary cohort (41 of 118 [34.7%] vs. 30 of 52 [57.7%], *p* = 0.005), whereas other characteristics were not significantly different between the two cohorts. In the primary cohort, 37 (31.3%) and 53 (44.9%) of the 118 HCC patients had MTM subtype and high Ki-67 pattern, respectively. Furthermore, 28 of the 37 MTM-HCCs and 26 of the non-MTM-HCCs were positive for high Ki-67 pattern (*p* < 0.001, Fig. 2a). Patients with MTM-HCC were prone to have preoperative serum α-fetoprotein (AFP) > 20 ng/mL (23 of 37 [62.2%] vs. 22 of 81 [27.2%], *p* < 0.001), AFP-L3% > 10 U/mL (20 of 37 [54.0%] vs. 21 of 81 [25.9%], *p* = 0.013), larger main tumor size (mean, 6.1 cm ± 3.2 [SD] vs. 4.6 cm ± 2.2 [SD], *p* = 0.015), Edmonson-Steiner grade III or IV tumor (25 of 37 [67.5%] vs. 5 of 81 [6.1%], *p* < 0.001), macrovascular invasion (3 of 37 [8.1%] vs. 0 of 81 [0%], *p* = 0.029) and microvascular invasion (22 of 37 [59.5%] vs. 14 of 81 [17.2%], *p* < 0.001, Fig. 2b) respectively, compared to patients with non-MTM-HCCs (Table 2). In parallel, in comparison to HCC patients with low Ki-67 pattern, HCC patients with high Ki-67 pattern appeared to be younger (mean, 59 years ± 9 [SD] vs. 63 years ± 11 [SD], *p* = 0.030), with more preoperative serum AFP > 20 ng/mL (26 of 53 [49.1%] vs. 19 of 65 [29.2%], *p* = 0.027), AFP-L3% > 10 U/mL (27 of 53 [50.9%] vs. 14 of 65 [21.5%], *p* ≤ 0.001), larger main tumor size (mean, 5.7 cm ± 3.2 [SD] vs. 4.6 cm ± 2.0 [SD], *p* = 0.028), Edmonson-Steiner grade III or IV tumor (25 of 53 [47.1%] vs. 5 of 65 [7.6%], *p* < 0.001) and microvascular invasion (23 of 53 [43.3%] vs. 13 of 65 [20.0%], *p* = 0.006, Fig. 2c), respectively.

**Table 1:** Clinical and pathologic characteristics of HCC patients in the primary and validation cohorts

Variables	Primary cohort (*n* = 118)	Validation cohort (*n* = 52)	*p*-value
Age (years)	61 ± 10	62 ± 11	0.486
Male sex	88 (72.7)	42 (80.8)	0.380
Etiology			0.399
HBV	89 (73)	36 (69.2)	
Others	29 (24.5)	16 (30.8)	
Preoperative tumor marker			
AFP > 20 ng/mL	45 (38.1)	28 (53.8)	0.057
AFP-L3% > 10 U/mL	41 (34.7)	30 (57.7)	0.005
DCP > 40 U/mL	92 (77.9)	45 (86.5)	0.193
Child-Pugh class			1.000
A	116 (98.3)	51 (98.1)	
B or C	2 (1.6)	1 (1.9)	
Main tumor size (cm)	5.1 ± 2.7	4.6 ± 2.0	0.245
No. of lesions			0.912
Solitary	106 (89.8)	47 (90.4)	
Multiple	12 (10.1)	5 (9.6)	
Edmonson-Steiner grade			0.744
I–II	88 (74.5)	40 (76.9)	
III-IV	30 (25.4)	12 (23.1)	
Cirrhosis	65 (55.0)	27 (51.9)	0.703
Satellite nodules	3 (2.5)	4 (7.7)	0.202
Macrovascular invasion	3 (2.5)	2 (3.8)	0.642
Microvascular invasion	36 (30.5)	20 (38.5)	0.309
Capsular invasion	61 (51.6)	27 (51.9)	0.978
MTM-HCC subtype	37 (31.3)	10 (19.2)	0.103
High Ki-67 pattern	53 (44.9)	31 (59.6)	0.077

Data are the number of patients, and data in parentheses are percentages. Mean values are ± standard deviations

*HCC* hepatocellular carcinoma, *HBV* hepatitis B virus, *AFP* α-fetoprotein, *AFP-L3%* lens culinaris agglutinin-bound fraction of α-fetoprotein, *DCP* des-gamma-carboxy prothrombin, *MTM* macrotrabecular-massive

**Table 2:** Clinical and pathologic characteristics of HCC patients in the primary cohorts based on MTM subtype and high Ki-67 pattern

Variables	MTM (*n* = 37)	Non-MTM (*n* = 81)	*p*-value^a^	High Ki-67 (*n* = 53)	Low Ki-67 (*n* = 65)	*p*-value^b^
Age (years)	60 ± 10	62 ± 11	0.408	59 ± 9	63 ± 11	0.030
Male sex	27 (72.9)	61 (75.3)	0.787	39 (73.5)	49 (75.3)	0.823
Etiology			0.614			0.384
HBV	29 (78.3)	60 (74.0)		42 (79.2)	47 (72.3)	
Others	8 (21.6)	21 (25.9)		11 (20.7)	18 (27.6)	
Preoperative marker						
AFP > 20 ng/mL	23 (62.2)	22 (27.2)	< 0.001	26 (49.1)	19 (29.2)	0.027
AFP-L3% > 10 U/mL	20 (54.0)	21 (25.9)	0.013	27 (50.9)	14 (21.5)	< 0.001
DCP > 40 U/mL	28 (75.6)	64 (79.0)	0.685	42 (79.2)	50 (76.9)	0.762
Child-Pugh class			0.567			0.884
A	36 (97.2)	80 (98.7)		52 (98.1)	64 (98.4)	
B or C	1 (2.7)	1 (1.2)		1 (1.8)	1 (1.5)	
Main tumor size (cm)	6.1 ± 3.2	4.6 ± 2.2	0.015	5.7 ± 3.2	4.6 ± 2.0	0.028
No. of lesions			0.417			0.324
Solitary	32 (86.4)	74 (91.3)		46 (86.7)	60 (92.3)	
Multiple	5 (13.5)	7 (8.6)		7 (13.2)	5 (7.6)	
Edmonson-Steiner grade			< 0.001			< 0.001
I–II	12 (32.4)	76 (93.8)		28 (52.8)	60 (92.3)	
III-IV	25 (67.5)	5 (6.1)		25 (47.1)	5 (7.6)	
Cirrhosis	22 (59.4)	43 (53.0)	0.518	32 (60.3)	33 (50.7)	0.297
Satellite nodules	2 (5.4)	1 (1.2)	0.182	3 (5.6)	0 (0)	0.088
Macrovascular invasion	3 (8.1)	0 (0)	0.029	1 (1.8)	2 (3.1)	1.000
Microvascular invasion	22 (59.5)	14 (17.2)	< 0.001	23 (43.3)	13 (20.0)	0.006
Capsular invasion	23 (62.1)	38 (46.9)	0.124	26 (49.0)	35 (53.8)	0.605

Data are the number of patients, and data in parentheses are percentages. Mean values are ± standard deviations

*HCC* hepatocellular carcinoma, *MTM* macrotrabecular-massive, *HBV* hepatitis B virus, *AFP* α-fetoprotein, *AFP-L3%* lens culinaris agglutinin-bound fraction of α-fetoprotein, *DCP* des-gamma-carboxy prothrombin

^a^ *p*-values comparing MTM-HCC with non-MTM-HCC

^b^ *p*-values comparing high Ki-67-expressed HCC with low Ki-67-expressed HCC

### B-mode US and CEUS features

**Fig. 3:**
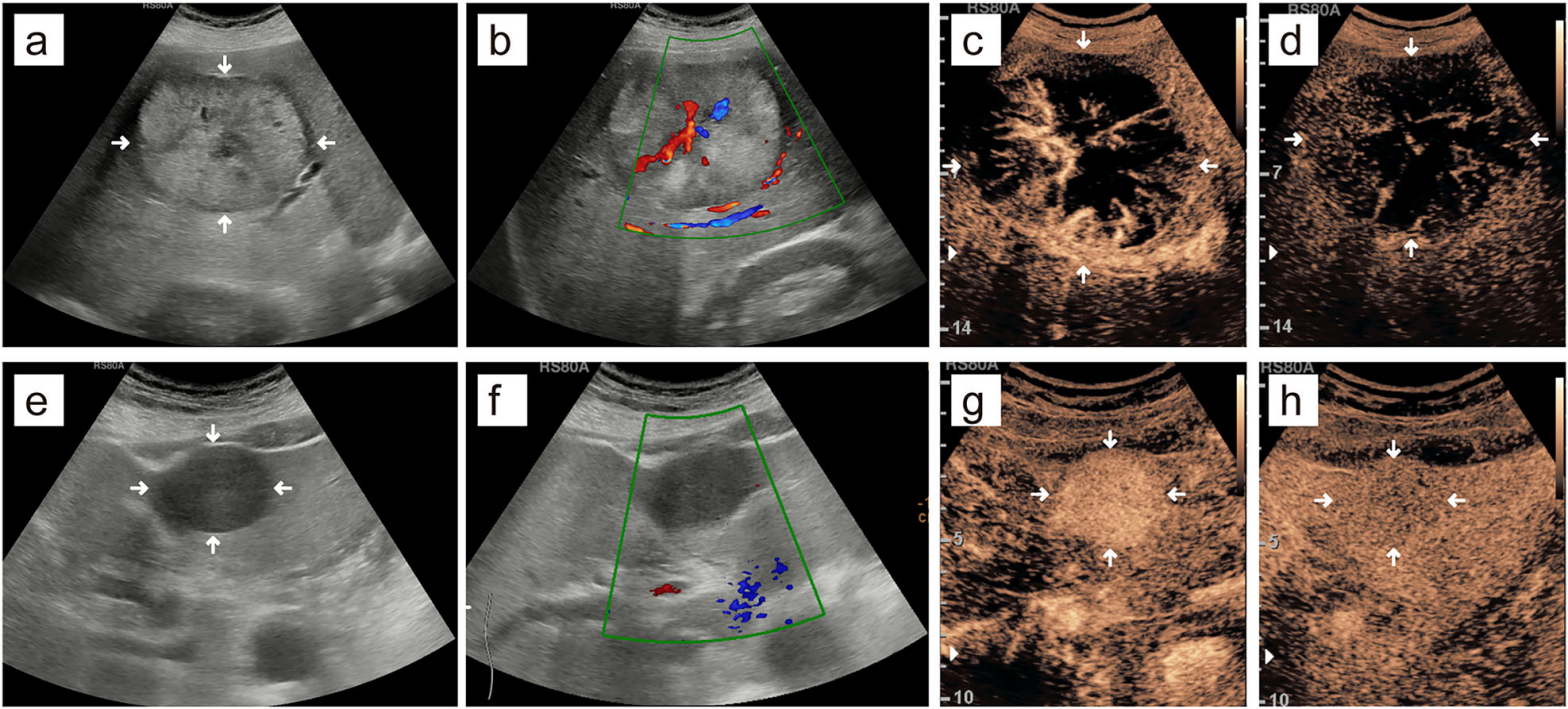
**a**–**d** Typical US/CEUS images in a 50-year-old man with hepatitis B virus-related HCC, positive for both MTM-HCC subtype and high Ki-67 pattern. **a** B-mode US showed a 10.3 cm isoechoic HCC lesion with halo sign located in the right liver lobe. **b** CDFI showed the intratumoral artery and peritumoral nutrient vessel. **c** Arterial phase branch-like heterogeneous hyperenhancement with a large area of nonenhancement was observed at 20 s after contrast agent injection. **d** Portal vein phase marked hypo-enhancement was observed at 101 s after contrast agent injection. **e**–**h** Typical US/CEUS images in a 72-year-old man with autoimmune hepatitis-related HCC, negative for both MTM-HCC subtype and high Ki-67 pattern. **e** B-mode US showed a 4.0 cm hypoechoic HCC lesion located in the left liver lobe. **f** CDFI showed no significant colored blood flow. **g** Arterial phase homogeneous hyperenhancement was observed at 20 s after contrast agent injection. **h** Portal vein phase iso-enhancement was observed at 100 s after contrast agent injection. US, ultrasound; CEUS, contrast-enhanced ultrasound; HCC, hepatocellular carcinoma; MTM, macrotrabecular-massive; CDFI, color Doppler flow imaging

MTM-HCCs were more often larger than 5 cm (19 of 37 [51.4%] vs. 27 of 81 [33.3%], *p* = 0.047), intratumoral necrosis (21 of 37 [56.8%] vs. 26 of 81 [32.1%], *p* = 0.011), intratumoral artery (14 of 37 [37.8%] vs. 14 of 81 [17.3%], *p* = 0.015), peritumoral nutrient vessel (23 of 37 [62.2%] vs. 32 of 81 [39.5%], *p* = 0.022), AP heterogeneous enhancement (33 of 37 [89.2%] vs. 55 of 81 [67.9%], *p* = 0.013), AP branched enhancement pattern (10 of 37 [27.0%] vs. 7 of 81 [11.1%], *p* = 0.029) and PVP hypo-/marked hypo-enhancement (27 of 37 [73.0%] vs. 41 of 81 [50.6%], *p* = 0.023), respectively, compared to non-MTM-HCCs (Table 3 and Fig. 3). Compared to HCCs with low Ki-67 pattern, HCCs with high Ki-67 pattern were more frequently intratumoral necrosis (27 of 53 [50.9%] vs. 20 of 65 [30.8%], *p* = 0.026) and AP branched enhancement pattern (14 of 53 [26.4%] vs. 5 of 65 [7.7%], *p* = 0.006) (Table 3 and Fig. 3). The interobserver consistency for B-mode US and CEUS features ranged from moderate to excellent (κ = 0.658–0.931) and the intra-observer agreement analysis was excellent (κ = 0.853–0.983) (Table S1). There was no obvious difference between MTM-HCCs and non-MTM-HCCs (*p* = 0.128) or HCCs with high Ki-67 pattern and HCCs with low Ki-67 pattern (*p* = 0.281) in the CEUS LI-RADS version 2017 category.

**Table 3:** The B-mode ultrasound and CEUS features of HCC patients in the primary cohorts based on MTM subtype and high Ki-67 pattern

Variables	MTM (*n* = 37)	Non-MTM (*n* = 81)	*p*-value^a^	High Ki-67 (*n* = 53)	Low Ki-67 (*n* = 65)	*p*-value^b^
Mean tumor size > 5 cm	19 (51.4)	27 (33.3)	0.047	25 (47.2)	21 (32.3)	0.100
Right lobe involvement	27 (72.9)	61 (75.3)	0.787	40 (75.4)	48 (73.8)	0.840
Echogenicity			0.254			0.883
Hyper-/iso-	22 (59.5)	39 (48.1)		27 (50.9)	34 (52.3)	
Hypo-	15 (40.5)	42 (51.9)		26 (49.1)	31 (47.7)	
Heterogeneous echo	32 (86.5)	72 (88.9)	0.708	47 (88.7)	57 (87.7)	0.869
Irregular shape	34 (91.9)	74 (91.4)	1.000	50 (94.3)	58 (89.2)	0.510
Ill-defined margin	35 (94.6)	80 (98.8)	0.231	52 (98.1)	63 (96.9)	1.000
Blood flow signal	35 (94.6)	65 (80.2)	0.054	44 (83.0)	56 (86.2)	0.638
Halo sign	14 (37.8)	30 (37.0)	0.933	18 (34.0)	26 (40.0)	0.500
Intratumoral necrosis	21 (56.8)	26 (32.1)	0.011	27 (50.9)	20 (30.8)	0.026
Intratumoral artery	14 (37.8)	14 (17.3)	0.015	17 (32.1)	11 (16.9)	0.054
Peritumoral nutrient vessel	23 (62.2)	32 (39.5)	0.022	29 (54.7)	26 (40.0)	0.111
AP heterogeneous enhancement	33 (89.2)	55 (67.9)	0.013	43 (81.1)	45 (69.2)	0.140
AP enhancement capsule	5 (13.5)	4 (4.9)	0.137	7 (13.2)	2 (3.1)	0.076
AP branched enhancement	10 (27.0)	9 (11.1)	0.029	14 (26.4)	5 (7.7)	0.006
PVP enhancement degree			0.023			0.864
Hyper-/iso-	10 (27.0)	40 (49.4)		22 (41.5)	28 (43.1)	
Hypo-/marked hypo-	27 (73.0)	41 (50.6)		31 (58.5)	37 (56.9)	
LP enhancement degree			0.307			0.626
Iso-	0 (0)	4 (4.9)		1 (1.9)	3 (4.6)	
Hypo-/marked hypo-	37 (100)	77 (95.1)		52 (98.1)	62 (95.4)	
CEUS LI-RADS version 2017 category			0.128			0.281
LR-4	0 (0)	1 (1.2)		0 (0)	1 (1.5)	
LR-5	27 (73.0)	65 (80.2)		45 (84.9)	47 (72.3)	
LR-M	8 (5.4)	15 (18.5)		7 (13.2)	16 (24.6)	
LR-TIV	2 (21.6)	0 (0)		1 (1.9)	1 (1.5)	

Data are the number of patients, and data in parentheses are percentages

*CEUS* contrast-enhanced ultrasound, *HCC* hepatocellular carcinoma, *MTM* macrotrabecular-massive, *AP* arterial phase, *PVP* portal vein phase, *LP* late phase, *LI-RADS* Liver Imaging Reporting and Data System

^a^ *p*-values comparing MTM-HCC with non-MTM-HCC

^b^ *p*-values comparing high Ki-67-expressed HCC with low Ki-67-expressed HCC

### Dynamic CEUS quantitative analysis

In comparison to the dynamic CEUS quantitative parameters of non-MTM-HCCs, MTM-HCCs revealed higher tumor MeanLin ratio, margin MeanLin ratio, margin PE ratio, margin WiAUC ratio, margin WiPI ratio, margin WoAUC ratio and margin WiWoAUC ratio (*p* < 0.001 for all) (Table S2). Compared to HCCs with low Ki-67 pattern, high Ki-67-expressed HCC showed higher tumor MeanLin ratio, tumor PE ratio, tumor WiPI ratio, margin MeanLin ratio, margin PE ratio, margin WiAUC ratio, margin WiR ratio, margin WiPI ratio, margin WoR ratio (*p* < 0.001 for all) (Table S2). Based on the ROC analysis for the primary cohort, the optimal cutoff value for MTM-HCC prediction was estimated to be greater than or equal to 3.96, 1.43, 1.55, 1.41, 1.50, 1.32 and 1.32 for tumor MeanLin ratio, margin MeanLin ratio, margin PE ratio, margin WiAUC ratio, margin WiPI ratio, margin WoAUC ratio and margin WiWoAUC ratio, respectively (Table S3). While for high Ki-67 pattern identification, the optimal cutoff value was estimated to be greater than or equal to 2.98, 3.52, 6.52, 1.75, 2.21, 1.54, 2.05, 2.55 and 2.29 for tumor MeanLin ratio, tumor PE ratio, tumor WiPI ratio, margin MeanLin ratio, margin PE ratio, margin WiAUC ratio, margin WiR ratio, margin WiPI ratio, margin WoR ratio, respectively (Table S3).

### Predictive models for MTM subtype and high Ki-67 pattern

**Fig. 4:**
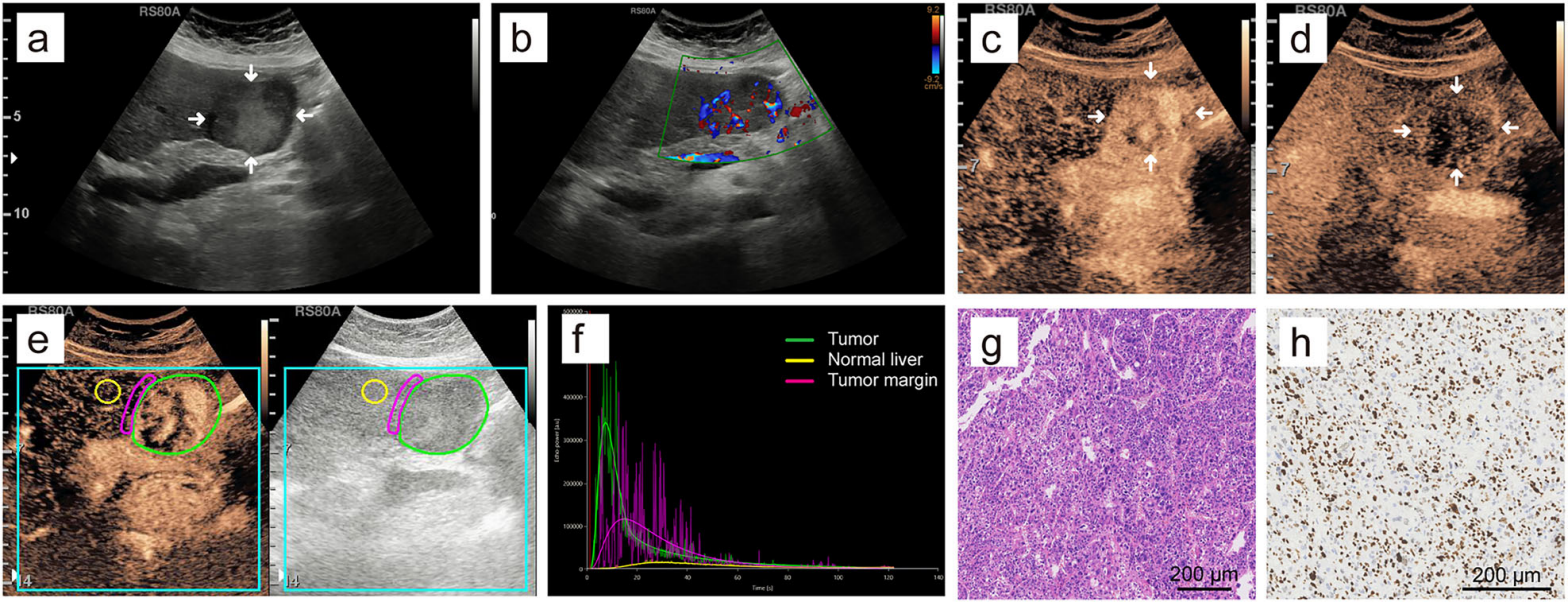
Images in a 58-year-old woman with hepatitis B virus-related HCC and baseline serum AFP level of 117 ng/mL. HCC was positive for both MTM-HCC subtype and high Ki-67 expression pattern. **a** B-mode ultrasound showed a 5.1 cm HCC located in the left liver lobe. **b** Color Doppler flow imaging revealed abundant dotted-linear colored blood flow signals. **c** Arterial phase heterogeneous hyperenhancement with a small area of nonenhancement was observed at 21 s after contrast agent injection. **d** Portal vein phase hypo-enhancement was observed at 90 s after contrast agent injection. **e** D-CEUS perfusion analysis exhibited the outlined ROIs, including HCC lesion (green), normal liver parenchyma (yellow) and HCC margin (magenta). **f** Time intensity curves automatically generated based on ROIs. **g** Hematoxylin-eosin staining showed distinct MTM-HCC appearance with trabeculae of over six cells thick surrounded by vascular spaces (original magnification × 10). **h** Immunohistochemistry showed high Ki-67 expression pattern (Ki-67^+^, 60%) (original magnification × 10). HCC, hepatocellular carcinoma; AFP, α-fetoprotein; MTM, macrotrabecular-massive; D-CEUS, dynamic contrast-enhanced ultrasound; ROIs, regions of interest

**Fig. 5:**
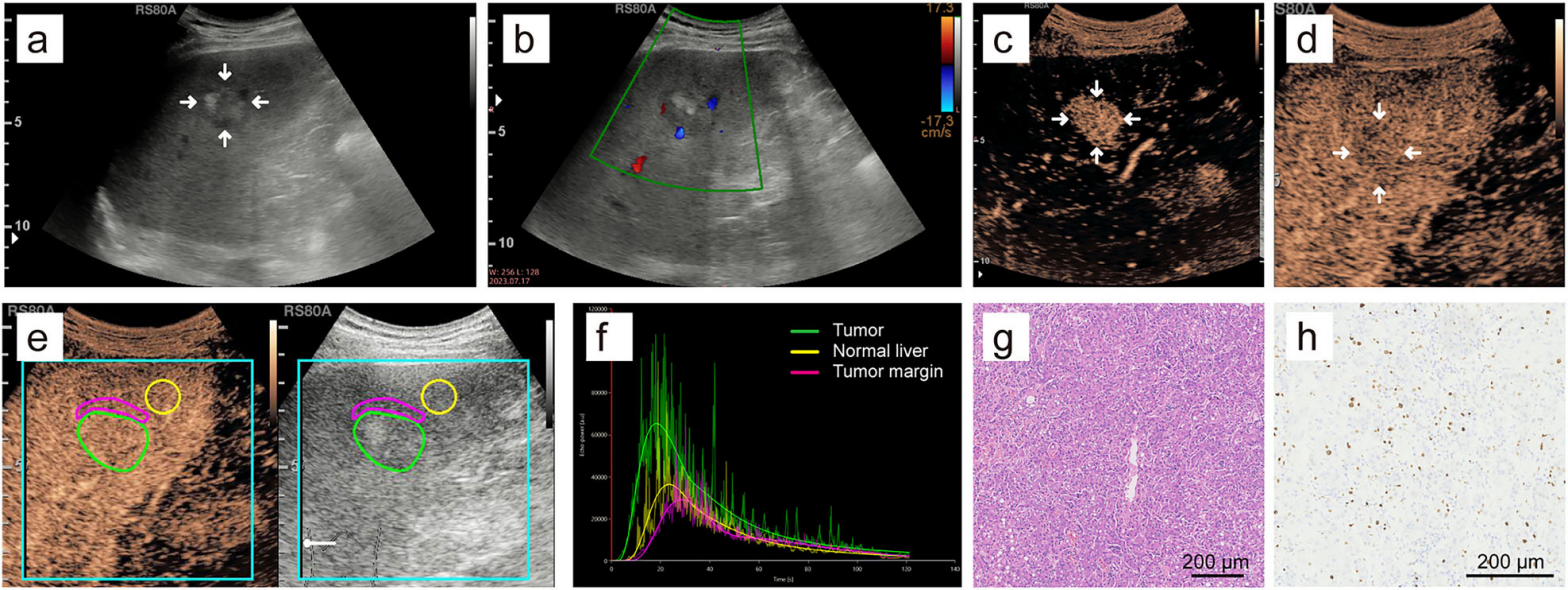
Images in a 64-year-old man with hepatitis B virus-related HCC and baseline serum AFP level of 4.8 ng/mL. HCC was negative for both MTM-HCC subtype and high Ki-67 expression pattern. **a** B-mode ultrasound showed a 2.6 cm HCC located in the right liver lobe. **b** Color Doppler flow imaging revealed a few dotted colored blood flow signals. **c** Arterial phase homogeneous hyperenhancement was observed at 19 s after contrast agent injection. **d** Portal vein phase iso-enhancement was observed at 93 s after contrast agent injection. **e** D-CEUS perfusion analysis exhibited the outlined ROIs, including HCC lesion (green), normal liver parenchyma (yellow) and HCC margin (magenta). **f** Time intensity curves automatically generated based on ROIs. **g** Hematoxylin-eosin staining showed microtrabecular feature (original magnification × 10). **h** Immunohistochemistry showed low Ki-67 expression pattern (Ki-67^+^, 5%) (original magnification × 10). HCC, hepatocellular carcinoma; AFP, α-fetoprotein; MTM, macrotrabecular-massive; D-CEUS, dynamic contrast-enhanced ultrasound; ROIs, regions of interest

By combining clinical data and US/CEUS features with multivariable logistic regression equation, Clinic-CEUS models were constructed for the prediction of MTM subtype (variables: AFP level, intratumoral necrosis, peritumoral nutrient vessel) and high Ki-67 pattern (variables: age, AFP-L3% level, AP branched enhancement), respectively (Table S4). The cutoff values of the Clinic-CEUS model derived from the primary cohort were 0.25 and 0.32 for MTM subtype and high Ki-67 pattern, respectively (Table S5). Further multivariable logistic regression analysis displayed that serum AFP > 20 ng/mL (odds ratio (OR) = 4.2; 95% CI: 1.5–11.7; *p* = 0.007), PVP hypo-/marked hypo-enhancement (OR = 3.6; 95% CI: 1.2–10.5; *p* = 0.019), tumor MeanLin ratio ≥ 3.96 (OR = 7.1; 95% CI: 2.4–20.7; *p* < 0.001) and margin MeanLin ratio ≥ 1.43 (OR = 4.4; 95% CI: 1.3–14.9; *p* = 0.018) were independently related to MTM subtype, whereas age less than 59 years (OR = 3.1; 95% CI: 1.2–7.8; *p* = 0.016), serum AFP-L3% > 10 U/mL (OR = 3.1; 95% CI: 1.2–8.0; *p* = 0.022), tumor MeanLin ratio ≥ 2.98 (OR = 2.9; 95% CI: 1.0, 8.2; *p* = 0.049) and margin WiPI ratio ≥ 2.55 (OR = 4.8; 95% CI: 1.6, 13.8; *p* = 0.004) were independently associated with high Ki-67 pattern in the primary cohort (Table 4 and Figs. 4, 5). By combining these predictors with logistic regression equation, Clinic-Q-CEUS model were constructed for the prediction of MTM subtype (variables: AFP level, PVP enhancement degree, tumor MeanLin ratio and margin MeanLin ratio) and high Ki-67 pattern (variables: age, AFP-L3% level, tumor MeanLin ratio and margin WiPI ratio), respectively (Table 4). The cutoff values of Clinic-Q-CEUS model derived from the primary cohort were 0.22 and 0.39 for MTM subtype and high Ki-67 pattern, respectively, which were then applied in the validation cohort for performance assessment (Table S5).

**Table 4:** Predictors of Clinic-Q-CEUS models for MTM subtype and high Ki-67 pattern by logistic regression analysis in primary cohort

Variables	Univariable OR	*p*-value^a^	Multivariable OR	*p*-value^b^
MTM-HCC subtype				
Serum AFP > 20 ng/mL	4.4 (1.9, 10.1)	< 0.001	4.2 (1.5, 11.7)	0.007
AFP-L3% > 10 U/mL	3.4 (1.5, 7.6)	0.004		0.435
Mean tumor size > 5 cm	2.1 (1.0, 4.7)	0.065		0.702
Intratumoral necrosis	2.8 (1.2, 6.2)	0.012		0.173
Intratumoral artery	2.9 (1.2, 7.0)	0.017		0.646
Peritumoral nutrient vessel	2.5 (1.1, 5.6)	0.024		0.195
AP heterogeneous enhancement	3.9 (1.3, 12.2)	0.019		0.272
AP branched enhancement	3.0 (1.1, 8.1)	0.034		0.643
PVP enhancement degree (Hypo-/marked hypo-)	2.6 (1.1, 6.1)	0.025	3.6 (1.2, 10.5)	0.019
Tumor MeanLin ratio ≥ 3.96	7.8 (3.3, 18.8)	< 0.001	7.1 (2.4, 20.7)	< 0.001
Margin MeanLin ratio ≥ 1.43	7.6 (2.7, 21.5)	< 0.001	4.4 (1.3, 14.9)	0.018
Margin PE ratio ≥ 1.55	4.9 (1.9, 12.3)	< 0.001		0.506
Margin WiAUC ratio ≥ 1.41	5.6 (2.2, 14.3)	< 0.001		0.802
Margin WiPI ratio ≥ 1.50	5.3 (2.0, 14.1)	< 0.001		0.476
Margin WoAUC ratio ≥ 1.32	5.8 (2.2, 15.5)	< 0.001		0.458
Margin WiWoAUC ratio ≥ 1.32	8.0 (2.8, 22.6)	< 0.001		0.526
High Ki-67 pattern				
Age < 59 years	3.0 (1.4, 6.4)	0.004	3.1 (1.2, 7.8)	0.016
Serum AFP > 20 ng/mL	2.3 (1.1, 5.0)	0.029		0.996
AFP-L3% > 10 U/mL	3.8 (1.7, 8.4)	0.001	3.1 (1.2, 8.0)	0.022
Intratumoral necrosis	2.3 (1.1, 5.0)	0.027		0.409
AP branched enhancement	4.3 (1.4, 12.9)	0.009		0.112
Tumor MeanLin ratio ≥ 2.98	4.8 (2.1, 10.9)	< 0.001	2.9 (1.0, 8.2)	0.049
Tumor PE ratio ≥ 3.52	3.9 (1.7, 9.0)	< 0.001		0.564
Tumor WiPI ratio ≥ 6.52	3.3 (1.5, 7.1)	< 0.001		0.239
Margin MeanLin ratio ≥ 1.75	5.4 (2.4, 12.3)	< 0.001		0.449
Margin PE ratio ≥ 2.51	4.8 (2.1, 10.8)	< 0.001		0.920
Margin WiAUC ratio ≥ 1.54	4.9 (2.2, 11.0)	< 0.001		0.974
Margin WiR ratio ≥ 2.05	5.5 (2.0, 15.1)	< 0.001		0.317
Margin WiPI ratio ≥ 2.55	5.7 (2.4, 13.2)	< 0.001	4.8 (1.6, 13.8)	0.004
Margin WoR ratio ≥ 2.55	5.9 (2.3, 15.3)	< 0.001		0.155

Data in parentheses are 95% CIs

*Clinic-Q-CEUS model* logistic regression model based on clinical data, CEUS features and dynamic CEUS quantitative parameters, *MTM-HCC* macrotrabecular-massive hepatocellular carcinoma, *OR* odds ratio, *AFP* α-fetoprotein, *AFP-L3%* lens culinaris agglutinin-bound fraction of α-fetoprotein, *AP* arterial phase, *PVP* portal vein phase, *MeanLin* average contrast signal intensity, *PE* peak enhancement, *WiAUC* wash-in area under the curve, *WiPI* wash-in perfusion index, *WoAUC* wash-out AUC, *WiWoAUC* wash-in and wash-out AUC, *WiR* wash-in rate, *WoR* wash-out rate

^a^ *p*-values for univariable odds ratio

^b^ *p*-values for multivariable odds ratio

### Diagnostic performances

**Fig. 6:**
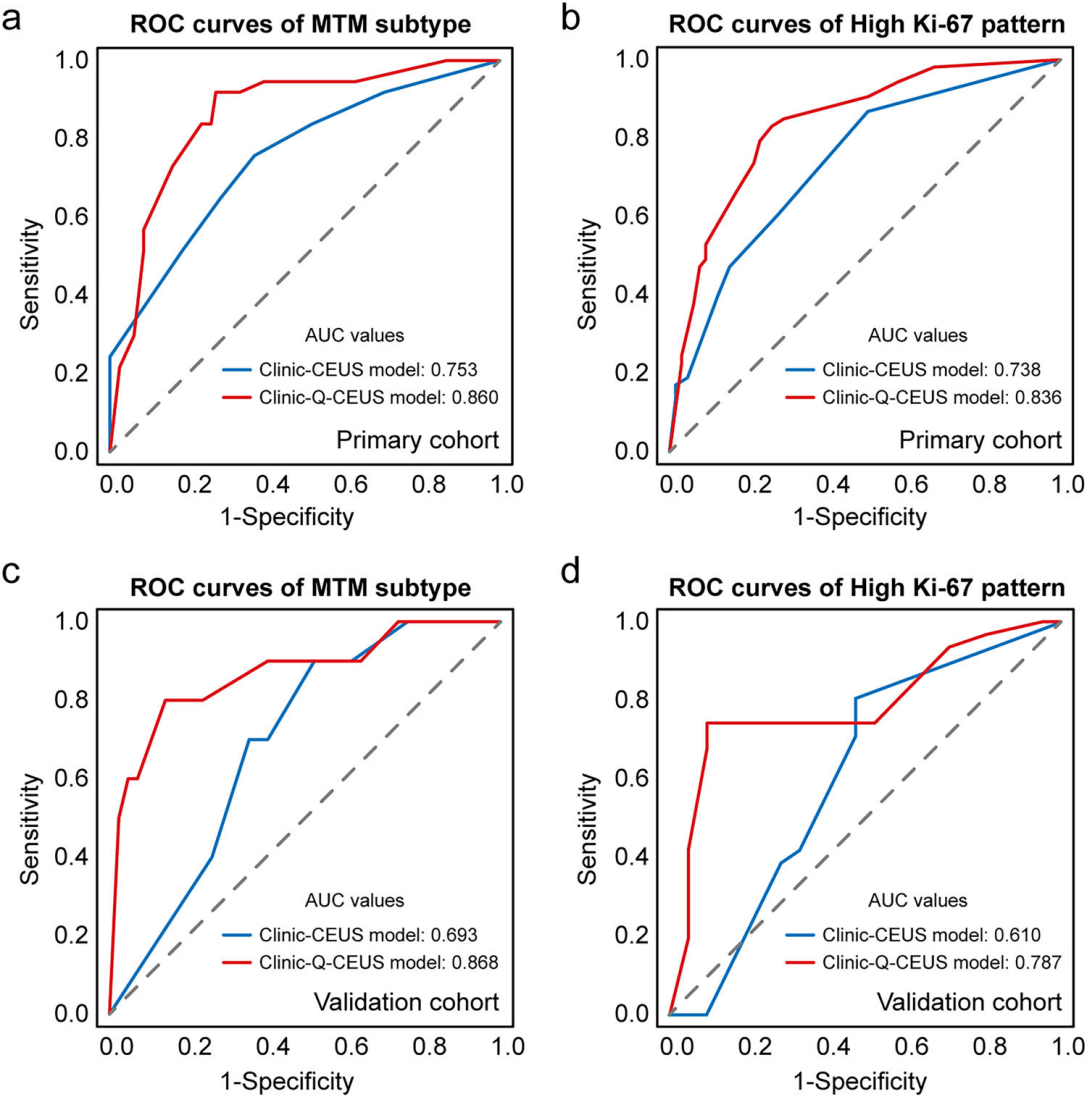
The receiver operating characteristic curves of the MTM subtype and high Ki-67 pattern in the primary cohort (**a**, **b**) and validation cohort (**c**, **d**), respectively. ROC, receiver operating characteristic; MTM, macrotrabecular-massive; AUC, area under curve; Clinic-CEUS model, logistic regression model based on clinical data and contrast-enhanced ultrasound (CEUS) features; Clinic-Q-CEUS model, logistic regression model based on clinical data, CEUS features and dynamic CEUS quantitative parameters

The diagnostic performances of the aforementioned regression models were illustrated in Table 5 and Fig. 6. The Clinic-Q-CEUS model had greater discrimination capacity for MTM subtype and high Ki-67 pattern than the Clinic-CEUS model in both the primary cohort (AUC: 0.860 vs. 0.753; *p* = 0.027 and 0.836 vs. 0.738; *p* = 0.036) and validation cohort (AUC: 0.868 vs. 0.693; *p* = 0.046 and 0.787 vs. 0.610, *p* = 0.018).

**Table 5:** Diagnostic performances of different regression-based predictive models

Predictive model	Sensitivity	Specificity	PPV	NPV	Accuracy	AUC (95% CI)
Primary cohort (MTM subtype)						
Clinic-CEUS model	0.514	0.815	0.559	0.786	0.720	0.753 (0.657, 0.850)
Clinic-Q-CEUS model	0.730	0.840	0.675	0.872	0.805	0.860 (0.788, 0.932)
* p*-value	0.039	0.791	/	/	0.076	0.027
Primary cohort (High Ki-67 pattern)						
Clinic-CEUS model	0.472	0.846	0.714	0.663	0.678	0.738 (0.651, 0.826)
Clinic-Q-CEUS model	0.660	0.831	0.761	0.750	0.754	0.836 (0.764, 0.909)
* p*-value	0.064	1.000	/	/	0.200	0.036
Validation cohort (MTM subtype)						
Clinic-CEUS model	0.400	0.738	0.267	0.838	0.673	0.693 (0.540, 0.846)
Clinic-Q-CEUS model	0.600	0.929	0.667	0.907	0.865	0.868 (0.725, 1.011)
* p*-value	1.000	0.012	/	/	0.013	0.046
Validation cohort (High Ki-67 pattern)						
Clinic-CEUS model	0.387	0.714	0.667	0.441	0.519	0.610 (0.444, 0.775)
Clinic-Q-CEUS model	0.742	0.905	0.920	0.704	0.808	0.787 (0.658, 0.917)
* p*-value	0.006	1.000	/	/	0.035	0.018

Data in parentheses are 95% CIs

*p*-values comparing Clinic-CEUS model with Clinic-Q-CEUS model

*PPV* positive predictive value, *NPV* negative predictive value, *AUC* area under the receiver operating characteristic curve, *CI* confidence interval, *MTM-HCC* macrotrabecular-massive hepatocellular carcinoma, *Clinic-CEUS model* logistic regression model based on clinical data and contrast-enhanced ultrasound (CEUS) features, *Clinic-Q-CEUS model* logistic regression model based on clinical data, CEUS features and dynamic CEUS quantitative parameters

## Discussion

MTM subtype and high Ki-67 pattern in HCC represent two aggressive types related to poor prognosis. The MTM subtype, first recognized by the 2019 5^th^ edition of the WHO classification, is characterized by prominent thick trabeculae (6–10 cells in thickness) with frequent vascular invasion [18]. MTM-HCC presents with relatively large tumor size, peritumoral satellite nodules, macro/microvascular invasion and biliary tract invasion [5]. Up to 60–70% MTM-HCC exhibit spontaneous intratumoral hemorrhage and necrosis on histology or imaging, likely due to dysregulated angiogenesis and insufficient vascular supply [19]. In particular, the Ki-67 proliferation index of MTM-HCC (~ 35–40%) was significantly higher than that of non-MTM-HCC (~ 15–20%), reflecting its hyperproliferative biology [20]. These features render MTM-HCC highly susceptible to early recurrence and poor prognosis [21]. The optimal clinical diagnosis and treatment regimen can be provided if MTM-HCC is accurately diagnosed preoperatively, as liver transplantation or radiofrequency ablation is not indicated for such patients, whereas transarterial chemoembolization combined with antiangiogenic therapy or immune checkpoint inhibitor-based immunotherapies is correlated with a longer disease-free survival [22–26]. Surgeons should perform anatomic hepatectomy or resection with wider margins, and recommend shorter follow-up intervals surveillance [27]. Therefore, preoperative noninvasive identification of these two aggressive subtypes may contribute to individualized treatment and improve patient prognosis.

In this study, we clarified that Serum AFP > 20 ng/mL, PVP hypo/marked hypo-enhancement, tumor MeanLin ratio ≥ 3.96 and margin MeanLin ratio ≥ 1.43 were independent predictors for MTM subtype, whereas age < 59 years, AFP-L3% > 10 U/mL, tumor MeanLin ratio ≥ 2.98 and margin WiPI ratio ≥ 2.55 were independent predictors for high Ki-67 pattern. We then developed Clinic-CEUS model and Clinic-Q-CEUS model for noninvasive prediction of both subtypes preoperatively based on clinical data and CEUS features with or without quantitative data, respectively. The Clinic-Q-CEUS model better predicted MTM subtype and high Ki-67 pattern than the Clinic-CEUS model with similar performance in the primary cohort and the validation cohort, indicating that dynamic CEUS quantitative analysis could serve as a valuable tool to provide important information for precision medical decision.

The incidence of MTM subtype and high Ki-67 pattern in our primary cohort was a bit higher than those reported in Luo et al [12] (MTM subtype, 16.7%) as well as Huang et al [28] (high Ki-67 pattern, 30%). This may be interpreted as the different main tumor size and the microvascular invasion status. Nevertheless, the outcomes of other studies also supported our findings [13, 19]. Our results showed that serum AFP level and PVP enhancement degree were independently related to MTM subtype. Previous studies have shown that MTM-HCC usually occurs in patients with hepatitis B infection and elevated serum AFP level [21]. The mostly PVP hypo/marked hypo-enhancement of MTM-HCC may correlate with its poorer differentiation, as studies have reported that low differentiated HCCs typically have fewer blood sinusoids and mainly feed from the hepatic artery [5, 29]. Our findings were also in agreement with the outcomes reported by Luo et al [12]. However, the comparatively narrow CIs for ORs estimated in our study should be ascribed to the higher prevalence of these subtypes. Meanwhile, our results displayed that age and serum AFP-L3% level were independently associated with a high Ki-67 pattern. The HCC patients with a high Ki-67 pattern are usually diagnosed earlier than those with a low Ki-67 pattern, which is probably due to the faster tumor proliferation leading to earlier onset of clinical symptoms in patients. AFP-L3 is derived from cancerous hepatocytes, and AFP-L3%-positive HCC tends to be poorly differentiated and more likely present with portal vein thrombosis [30].

Previous studies have shown that certain contrast-enhanced imaging features from contrast-enhanced computed tomography and contrast-enhanced magnetic resonance imaging can help to accurately diagnose aggressive subtypes of HCC. Of these, massive intratumoral necrosis, intratumor hemorrhage and AP hypo-enhancement components exceeding 20% or 50% have been recognized as independent predictors of MTM subtype, with a sensitivity of 0.65 to 0.88 and a specificity of 0.57 to 0.93, and predictive models combining the above indicators achieved diagnostic accuracy of 0.63 to 0.83 [8, 31, 32]. Moreover, irregular tumor margin, ill-defined pseudo-capsule and longer T1 relaxation time were found to be strongly correlated with the high Ki-67 pattern of HCC, with sensitivity, specificity and diagnostic accuracy ranging from 0.79 to 0.91, 0.67 to 0.90, and 0.78 to 0.93, respectively [33–35]. In our study, the Clinic-CEUS model integrates serum AFP, intratumoral necrosis and peritumoral nutrient vessel for MTM-HCC prediction and age, serum AFP-L3% and AP branched enhancement for high Ki-67 pattern identification, achieving an undesirable AUC of 0.720 and 0.673, respectively. While the high intra-observer agreement reflects the reproducibility of our standardized imaging criteria for US and CEUS features interpretations, the moderate interobserver consistency regarding irregular shape, ill-defined margin and AP heterogeneous enhancement features may reflect subjectivity in visual evaluation, emphasizing the value of applying quantitative tools.

Dynamic CEUS quantitative analysis allows more precise acquisition of parameters that indirectly reflect perfusion status to understand tumor angiogenesis and perfusion alterations. In this study, we reported for the first time the utility of Sonovue^®^-based CEUS quantitative parameters in the preoperative identification of MTM subtypes and high Ki-67 pattern. Our results indicated that the tumor MeanLin ratio and margin tumor MeanLin ratio, as well as tumor MeanLin ratio and margin WiPI ratio, were independent predictors for MTM subtype and high Ki-67 pattern, respectively. The AP hyperenhancement exhibited by HCC is owing to neoangiogenesis, causing a net increased arterial flow with concomitant increased permeability of immature vessels [36]. Angiogenesis activation is the main oncogenic pathway of MTM-HCC, with over-expressed angiogenic-2 and vascular endothelial growth factor coordinating with each other to promote angiogenesis [37]. The higher MeanLin ratio of tumor and margin in MTM-HCC may be explained by abundant intratumoral arteries and peritumoral nourishing vessels, which is consistent with the findings of Rhee et al [8]. Meanwhile, our study found that a high Ki-67 pattern occurred more frequently in MTM subtypes compared to non-MTM subtypes in the primary cohort. The Ki-67 level represents the active tumor cell proliferation degree, and a high Ki-67 pattern is closely linked to tumor invasiveness and metastasis [38]. Tumor invasion usually begins at the tumor margin and manifests as increased neovasculogenesis. The neovessels with larger lumens and better permeability allow for more contrast agents to enter tumor margin tissue, thus increased perfusion per unit of time [15]. Of note, none of the time-related quantitative parameters (e.g., RT, mTTl, TTP and FT) were statistically different between group comparisons. This may be influenced by factors such as the contrast agent injection speed and individual differences.

In our study, the Clinic-Q-CEUS model outperformed the Clinic-CEUS model substantially, either for identifying MTM subtype or high Ki-67 pattern. The Clinic-CEUS model simulates the decision-making process of a radiologist in a typical clinical situation based on the patient’s clinical information and CEUS features, while the Clinic-Q-CEUS model compensates for subjective interpretation bias of differently experienced radiologists by further integrating objective CEUS quantitative parameters. Our study demonstrated the value of CEUS quantitative parameters in the prediction of aggressive HCC subtypes, which represents a step toward the implementation of routine quantitative analysis into clinical practice and potentially provides guidance for selecting patients who may respond to existing targeted therapies.

Our study had several limitations. First, the findings were based on surgically resected tumors, which cannot represent the full spectrum of HCCs with inherent selection bias. Our results are currently applicable to surgical cohorts and need to be further validated in the future among patients with advanced diseases or those undergoing biopsy. Second, as the primary cohort was retrospectively performed and many cases were excluded due to poor DICOM videos, the diagnostic accuracy may potentially be overestimated. Third, since the primary cohort retrospectively collected data from the last two years and lacked follow-up time, the association between dynamic CEUS quantitative parameters and the overall prognosis of aggressive HCC subtypes deserves further exploration in the future. Fourth, the current study did not include cases with Sonozoid-based CEUS due to technical limitations, and specialized quantitative software will be needed in the future to analyze its unique pharmacokinetic behavior and to clarify whether the Kupffer phase can provide additional predictive information. Finally, the overall cohort had small sample sizes, and the efficacy of the model needs to be validated in large multicenter prospective study cohorts.

## Conclusion

In conclusion, quantitative parameters of dynamic CEUS could serve as a useful complement to clinical data and CEUS features for preoperative identification of MTM subtype and high Ki-67 pattern in HCC patients. The tumor MeanLin ratio and margin tumor MeanLin ratio, as well as tumor MeanLin ratio and margin WiPI ratio, are valuable predictors of MTM subtype and high Ki-67 pattern, respectively.

## Supplementary information

ELECTRONIC SUPPLEMENTARY MATERIAL

## Data Availability

The datasets used or analyzed during the current study are available from the corresponding author upon reasonable request.

## Abbreviations

AFP: α-Fetoprotein
AFP-L3%: Lens culinaris agglutinin-bound fraction of α-fetoprotein
AP: Arterial phase
CEUS: Contrast-enhanced ultrasound
CI: Confidence interval
DICOM: Digital imaging and communications in medicine
FT: Fall time
HCC: Hepatocellular carcinoma
LI-RADS: Liver Imaging Reporting and Data System
LP: Late phase
MeanLin: Average contrast signal intensity
MTM: Macrotrabecular-massive
mTTl: Mean transit time local
OR: Odds ratio
PE: Peak enhancement
PVP: Portal vein phase
RT: Rise time
SD: Standard deviations
TIC: Time intensity curves
TTP: Time to peak
WiAUC: Wash-in area under the curve
WiPI: Wash-in perfusion index
WiR: Wash-in rate
WiWoAUC: Wash-in and wash-out area under the curve
WoAUC: Wash-out area under the curve
WoR: Wash-out rate

## References

[1] Singal AG, Kanwal F, Llovet JM (2023) Global trends in hepatocellular carcinoma epidemiology: implications for screening, prevention and therapy. Nat Rev Clin Oncol 20:864–884. 10.1038/s41571-023-00825-3 37884736 10.1038/s41571-023-00825-3

[2] Vyas M, Zhang X (2020) Hepatocellular carcinoma: role of pathology in the era of precision medicine. Clin Liver Dis 24:591–610. 10.1016/j.cld.2020.07.010 33012447 10.1016/j.cld.2020.07.010

[3] Ziol M, Pote N, Amaddeo G et al (2018) Macrotrabecular-massive hepatocellular carcinoma: a distinctive histological subtype with clinical relevance. Hepatology 68:103–112. 10.1002/hep.29762 29281854 10.1002/hep.29762

[4] Luo Y, Ren F, Liu Y et al (2015) Clinicopathological and prognostic significance of high Ki-67 labeling index in hepatocellular carcinoma patients: a meta-analysis. Int J Clin Exp Med 8:10235–10247 26379815 PMC4565198

[5] Li X, Yao Q, Liu C et al (2022) Macrotrabecular-massive hepatocellular carcinoma: what should we know? J Hepatocell Carcinoma 9:379–387. 10.2147/JHC.S364742 35547829 10.2147/JHC.S364742 PMC9084381

[6] Bai K, Cao Y, Huang Q et al (2017) Prognostic value of Ki67 expression for patients with surgically resected hepatocellular carcinoma: perspectives from a high incidence area. Clin Lab 63:355–364. 10.7754/Clin.Lab.2016.160638 28182353 10.7754/Clin.Lab.2016.160638

[7] Li M, Fan Y, You H et al (2023) Dual-energy CT deep learning radiomics to predict macrotrabecular-massive hepatocellular carcinoma. Radiology 308:e230255. 10.1148/radiol.230255 37606573 10.1148/radiol.230255

[8] Rhee H, Cho ES, Nahm JH et al (2021) Gadoxetic acid-enhanced MRI of macrotrabecular-massive hepatocellular carcinoma and its prognostic implications. J Hepatol 74:109–121. 10.1016/j.jhep.2020.08.013 32818570 10.1016/j.jhep.2020.08.013

[9] Chen J, Chen C, Xia C et al (2018) Quantitative free-breathing dynamic contrast-enhanced MRI in hepatocellular carcinoma using gadoxetic acid: correlations with Ki67 proliferation status, histological grades, and microvascular density. Abdom Radiol (NY) 43:1393–1403. 10.1007/s00261-017-1320-3 28939963 10.1007/s00261-017-1320-3

[10] Zhao YM, Xie SS, Wang J et al (2023) Added value of CE-CT radiomics to predict high Ki-67 expression in hepatocellular carcinoma. BMC Med Imaging 23:138. 10.1186/s12880-023-01069-4 37737166 10.1186/s12880-023-01069-4 PMC10514983

[11] Park J, Lee JM, Kim TH et al (2022) Imaging diagnosis of hepatocellular carcinoma: future directions with special emphasis on hepatobiliary magnetic resonance imaging and contrast-enhanced ultrasound. Clin Mol Hepatol 28:362–379. 10.3350/cmh.2021.0361 34955003 10.3350/cmh.2021.0361 PMC9293611

[12] Luo M, Liu X, Yong J et al (2023) Preoperative prediction of macrotrabecular-massive hepatocellular carcinoma based on B-Mode US and CEUS. Eur Radiol 33:4024–4033. 10.1007/s00330-022-09322-0 36484835 10.1007/s00330-022-09322-0

[13] Zhang D, Zhang XY, Lu WW et al (2024) Predicting Ki-67 expression in hepatocellular carcinoma: nomogram based on clinical factors and contrast-enhanced ultrasound radiomics signatures. Abdom Radiol (NY) 49:1419–1431. 10.1007/s00261-024-04191-1 38461433 10.1007/s00261-024-04191-1

[14] Dietrich CF, Correas JM, Cui XW et al (2024) EFSUMB technical review—update 2023: dynamic contrast-enhanced ultrasound (DCE-CEUS) for the quantification of tumor perfusion. Ultraschall Med 45:36–46. 10.1055/a-2157-2587 37748503 10.1055/a-2157-2587

[15] Wiesinger I, Wiggermann P, Zausig N et al (2018) Percutaneous treatment of malignant liver lesions: evaluation of success using contrast-enhanced ultrasound (CEUS) and perfusion software. Ultraschall Med 39:440–447. 10.1055/s-0043-119353 28946152 10.1055/s-0043-119353

[16] Wiesinger I, Jung F, Jung EM (2021) Contrast-enhanced ultrasound (CEUS) and perfusion imaging using VueBox^®^. Clin Hemorheol Microcirc 78:29–40. 10.3233/CH-201040 33523044 10.3233/CH-201040

[17] Qian H, Shen Z, Zhou D et al (2023) Intratumoral and peritumoral radiomics model based on abdominal ultrasound for predicting Ki-67 expression in patients with hepatocellular cancer. Front Oncol 13:1209111. 10.3389/fonc.2023.1209111 37711208 10.3389/fonc.2023.1209111 PMC10498123

[18] Kim H, Jang M, Park YN (2020) Histopathological variants of hepatocellular carcinomas: an update according to the 5th edition of the WHO classification of digestive system tumors. J Liver Cancer 20:17–24. 10.17998/jlc.20.1.17 37383050 10.17998/jlc.20.1.17 PMC10035696

[19] Feng Z, Li H, Zhao H et al (2021) Preoperative CT for characterization of aggressive macrotrabecular-massive subtype and vessels that encapsulate tumor clusters pattern in hepatocellular carcinoma. Radiology 300:219–229. 10.1148/radiol.2021203614 33973839 10.1148/radiol.2021203614

[20] Calderaro J, Couchy G, Imbeaud S et al (2017) Histological subtypes of hepatocellular carcinoma are related to gene mutations and molecular tumour classification. J Hepatol 67:727–738. 10.1016/j.jhep.2017.05.014 28532995 10.1016/j.jhep.2017.05.014

[21] Loy LM, Low HM, Choi JY et al (2022) Variant hepatocellular carcinoma subtypes according to the 2019 WHO classification: an imaging-focused review. AJR Am J Roentgenol 219:212–223. 10.2214/AJR.21.26982 35170359 10.2214/AJR.21.26982

[22] Aggarwal A, Te HS, Verna EC et al (2021) A national survey of hepatocellular carcinoma surveillance practices following liver transplantation. Transplant Direct 7:e638. 10.1097/TXD.0000000000001086 33324743 10.1097/TXD.0000000000001086 PMC7725259

[23] Lee S, Kang TW, Song KD et al (2021) Effect of microvascular invasion risk on early recurrence of hepatocellular carcinoma after surgery and radiofrequency ablation. Ann Surg 273:564–571. 10.1097/SLA.0000000000003268 31058694 10.1097/SLA.0000000000003268

[24] Feng Z, Li H, Liu Q et al (2023) CT radiomics to predict macrotrabecular-massive subtype and immune status in hepatocellular carcinoma. Radiology 307:e221291. 10.1148/radiol.221291 36511807 10.1148/radiol.221291

[25] Kurebayashi Y, Matsuda K, Ueno A et al (2022) Immunovascular classification of HCC reflects reciprocal interaction between immune and angiogenic tumor microenvironments. Hepatology 75:1139–1153. 10.1002/hep.32201 34657298 10.1002/hep.32201

[26] Liu LL, Zhang SW, Chao X et al (2021) Coexpression of CMTM6 and PD-L1 as a predictor of poor prognosis in macrotrabecular-massive hepatocellular carcinoma. Cancer Immunol Immunother 70:417–429. 10.1007/s00262-020-02691-9 32770259 10.1007/s00262-020-02691-9 PMC7889680

[27] Zhang EL, Cheng Q, Huang ZY et al (2021) Revisiting surgical strategies for hepatocellular carcinoma with microvascular invasion. Front Oncol 11:691354. 10.3389/fonc.2021.691354 34123861 10.3389/fonc.2021.691354 PMC8190326

[28] Huang Z, Zhou P, Li S et al (2022) Prediction of the Ki-67 marker index in hepatocellular carcinoma based on dynamic contrast-enhanced ultrasonography with Sonazoid. Insights Imaging 13:199. 10.1186/s13244-022-01320-6 36536262 10.1186/s13244-022-01320-6 PMC9763522

[29] Chernyak V, Fowler KJ, Kamaya A et al (2018) Liver Imaging Reporting and Data System (LI-RADS) version 2018: imaging of hepatocellular carcinoma in at-risk patients. Radiology 289:816–830. 10.1148/radiol.2018181494 30251931 10.1148/radiol.2018181494 PMC6677371

[30] Kudo M (2013) Alpha-fetoprotein-L3: useful or useless for hepatocellular carcinoma? Liver Cancer 2:151–152. 10.1159/000343847 24400219 10.1159/000343847 PMC3881313

[31] Chen J, Xia C, Duan T et al (2021) Macrotrabecular-massive hepatocellular carcinoma: imaging identification and prediction based on gadoxetic acid-enhanced magnetic resonance imaging. Eur Radiol 31:7696–7704. 10.1007/s00330-021-07898-7 33856520 10.1007/s00330-021-07898-7

[32] Mule S, Galletto Pregliasco A, Tenenhaus A et al (2020) Multiphase liver MRI for identifying the macrotrabecular-massive subtype of hepatocellular carcinoma. Radiology 295:562–571. 10.1148/radiol.2020192230 32228294 10.1148/radiol.2020192230

[33] Qiu G, Chen J, Liao W et al (2023) Gadoxetic acid-enhanced MRI combined with T1 mapping and clinical factors to predict Ki-67 expression of hepatocellular carcinoma. Front Oncol 13:1134646. 10.3389/fonc.2023.1134646 37456233 10.3389/fonc.2023.1134646 PMC10348748

[34] Cai C, Wang L, Tao L et al (2025) Imaging-based prediction of Ki-67 expression in hepatocellular carcinoma: a retrospective study. Cancer Med 14:e70562. 10.1002/cam4.70562 39964132 10.1002/cam4.70562 PMC11834164

[35] Chen Y, Qin X, Long L et al (2020) Diagnostic value of Gd-EOB-DTPA-enhanced MRI for the expression of Ki67 and microvascular density in hepatocellular carcinoma. J Magn Reson Imaging 51:1755–1763. 10.1002/jmri.26974 31675163 10.1002/jmri.26974

[36] Yang ZF, Poon RT (2008) Vascular changes in hepatocellular carcinoma. Anat Rec 291:721–734. 10.1002/ar.20668 10.1002/ar.20668 18484619

[37] Calderaro J, Ziol M, Paradis V et al (2019) Molecular and histological correlations in liver cancer. J Hepatol 71:616–630. 10.1016/j.jhep.2019.06.001 31195064 10.1016/j.jhep.2019.06.001

[38] Lin YM, Taiji R, Calandri M et al (2021) Tumor biomarkers and interventional oncology: impact on local outcomes for liver and lung malignancy. Curr Oncol Rep 23:67. 10.1007/s11912-021-01056-4 33855606 10.1007/s11912-021-01056-4

