# Supplementary material for: Dynamic contrast-enhanced ultrasound perfusion analysis for preoperative prediction of aggressive hepatocellular carcinoma subtypes
Source: Insights Imaging. 2025 Sep 23;16:202. doi: 10.1186/s13244-025-02052-z (PMC12457242; doi:10.1186/s13244-025-02052-z)
Supplement: Supplementary file 1 — ELECTRONIC SUPPLEMENTARY MATERIAL [file 13244_2025_2052_MOESM1_ESM.pdf]

Dynamic Contrast-enhanced Ultrasound Perfusion Analysis for Preoperative Prediction of Aggressive Hepatocellular Carcinoma Subtypes

ELECTRONIC SUPPLEMENTARY MATERIAL

Materials and methods

Clinical information

Clinical data including age, sex, etiology, Child-Pugh class and preoperative serum indexes including AFP, AFP-L3% and DCP were recorded. For biomarkers that are not routinely covered by standard clinical regimen, such as AFP-L3% and DCP, patients enrolled in the study had signed a self-paid informed consent form during their hospitalization explicitly agreeing to cover this testing costs.

CEUS imaging acquisition

Patients at the Institute 1 underwent both US and CEUS examinations using Samsung RS80A (Samsung Ultrasound System, transducer CA1-7A, frequency range 1.0-7.0 MHz) by radiologists with 15 years of experience, and patients at the Institute 2 underwent US and CEUS examinations by using GE LogiqE9 (GE Healthcare, transducer C1-6, frequency range 1.5-6.0 MHz) by a radiologist with 10 years of experience. A low mechanical index of 0.08-0.12 was used for CEUS and the largest diameter section of target lesion was selected as the contrast plane as much as possible. 2 mL of SonoVue® contrast agents diluted in 0.9% saline was injected through the antecubital vein followed by a 5 mL saline flush. The CEUS operating mode and timer were started at the same time as the injection. Video clips were recorded continuously for at least 2 min, then scanned at 20-30 s intervals and recorded for 5 min or until the contrast agent disappeared. All imaging data were stored in DICOM format for further offline analysis.

Models construction

For Clinic-CEUS model construction, differences in clinical variables and US/CEUS imaging features related to MTM subtype and high Ki-67 pattern of HCC were compared by univariate logistic regression analyses. Candidates with $p < 0.05$ in the univariate analyses were retained for the next multivariable binary logistic regression to identify independent predictive parameters for aggressive HCC subtype. For Clinic-Q-CEUS model construction, differences in CEUS quantitative parameters associated

with MTM subtype and high Ki-67 pattern of HCC were analyzed by univariate logistic regression. Candidates with $p < 0.001$ were input into multi-variable binary logistic regression along with the previously selected clinical variables and US/CEUS imaging features to identify independent predictive parameters for aggressive HCC subtype. The final logistic regression model was expressed as:

$$\log\left(\frac{p}{1-p}\right) = \beta_0 + \beta_1 X_1 + \beta_2 X_2 + \cdots + \beta_n X_n$$

The p is the predictive probability and β_0 is the intercept. β_1 to β_n represents regression coefficients and X_1 to X_n denotes selected independent predictive parameters.

Table S1 Inter-observer and intra-observer agreement of US/CEUS features of HCCs in primary cohort.

	Inter-observer agreement	intra-observer agreement
US/CEUS features	κ	κ
Echogenicity	0.780	0.949
Irregular shape	0.684	0.866
Ill-defined margin	0.658	0.853
Blood flow signal	0.746	0.975
Halo sign	0.854	0.963
Intratumoral necrosis	0.789	0.982
Intratumoral artery	0.931	0.954
Peritumoral nutrient vessel	0.881	0.949
AP Heterogeneous enhancement	0.682	0.960
AP enhancement capsule	0.760	0.924
AP branched enhancement	0.846	0.911
PVP enhancement degree	0.931	0.983
LP enhancement degree	0.741	0.885

US, ultrasound; CEUS, contrast-enhanced ultrasound; HCC, hepatocellular carcinoma; AP, arterial phase; PVP, portal vein phase; LP, late phase.

Table S2 The dynamic CEUS quantitative parameters of HCC patients in the primary cohorts based on MTM subtype and high Ki-67 pattern.

Variables	MTM (n = 37)	Non-MTM (n = 81)	*p* value[a]	High Ki-67 (n = 53)	Low Ki-67 (n = 65)	*p* value[b]
MeanLin ratio						
Tumor	5.0 (5.0)	2.3 (2.7)	< 0.001	4.2 (4.8)	2.0 (2.7)	< 0.001
Margin	3.0 (3.9)	1.4 (1.7)	< 0.001	2.7 (3.0)	1.3 (0.9)	< 0.001
PE ratio						
Tumor	9.5 (14.4)	4.4 (5.7)	0.002	7.4 (11.2)	3.3 (5.2)	< 0.001
Margin	3.7 (6.8)	1.5 (2.2)	< 0.001	3.7 (4.6)	1.4 (1.2)	< 0.001
WiAUC ratio						
Tumor	3.8 (5.1)	1.7 (3.3)	0.005	3.8 (4.2)	1.7 (2.8)	0.002
Margin	2.4 (3.8)	1.2 (2.0)	< 0.001	2.2 (3.0)	1.1 (1.5)	< 0.001
RT (s)						
Tumor	8.6 (5.6)	8.4 (6.8)	0.761	8.6 (7.1)	8.5 (6.5)	0.644
Margin	16.5 (11.3)	17.5 (11.5)	0.700	16.4 (11.6)	18.5 (11.8)	0.078
mTTI (s)						
Tumor	35.1 (35.3)	40.7 (55.2)	0.151	32.6 (24.8)	53.4 (56.6)	0.001
Margin	75.0 (73.0)	78.1 (137.3)	0.337	64.5 (59.4)	112.7 (170.3)	0.001
TTP (s)						
Tumor	13.6 (8.1)	13.6 (6.8)	0.572	14.6 (8.4)	13.6 (6.5)	0.619
Margin	24.2 (11.5)	25.4 (15.2)	0.624	24.2 (14.3)	25.6 (13.6)	0.329
WiR ratio						
Tumor	12.1 (45.0)	8.5 (13.2)	0.008	16.5 (35.8)	7.2 (11.7)	0.002
Margin	4.4 (10.7)	2.1 (2.9)	0.009	4.9 (8.8)	1.8 (1.9)	< 0.001
WiPI ratio						
Tumor	7.3 (13.7)	4.2 (5.5)	0.002	7.3 (10.9)	3.3 (4.7)	< 0.001

Margin	3.7 (6.4)	1.5 (2.1)	< 0.001	3.6 (4.6)	1.3 (1.2)	< 0.001
WoAUC ratio						
Tumor	3.2 (4.5)	1.6 (3.2)	0.014	3.1 (3.9)	1.5 (3.0)	0.017
Margin	2.1 (3.6)	1.3 (1.7)	< 0.001	2.0 (2.5)	1.2 (1.9)	0.006
WiWoAUC ratio						
Tumor	3.3 (4.3)	1.6 (3.2)	0.013	3.3 (4.0)	1.5 (3.0)	0.010
Margin	2.1 (3.4)	1.3 (1.9)	< 0.001	2.1 (2.9)	1.2 (1.8)	0.002
FT (s)						
Tumor	17.3 (15.2)	16.5 (17.7)	0.837	16.1 (18.5)	17.7 (15.3)	0.308
Margin	36.5 (34.8)	39.8 (36.2)	0.864	32.1 (26.2)	47.3 (44.8)	0.010
WoR ratio						
Tumor	16.6 (59.0)	11.4 (25.1)	0.026	19.2 (54.5)	8.2 (22.6)	0.003
Margin	4.6 (15.8)	2.2 (5.4)	0.015	5.7 (14.7)	1.6 (3.5)	< 0.001

Data are median and data in parentheses are interquartile range.

[a] *p* values comparing MTM-HCC with non-MTM-HCC.

[b] *p* values comparing high Ki-67-expressed HCC with low Ki-67-expressed HCC.

CEUS, contrast-enhanced ultrasound; HCC, hepatocellular carcinoma; MTM, macrotrabecular-massive; MeanLin, average contrast signal intensity; PE, peak enhancement; WiAUC, wash-in area under the curve; RT, rise time; mTTI, mean transit time local; TTP, time to peak; WiR, wash-in rate; WiPI, wash-in perfusion index; WoAUC, wash-out AUC; WiWoAUC, wash-in and wash-out AUC; FT, fall time; WoR, wash-out rate.

Table S3 Converting dynamic CEUS quantitative parameters into binary variables.

Variables	AUROC	Youden index	Cutoff value	Value tending to MTM subtype
Tumor MeanLin ratio	0.755 (0.660, 0.850)	0.466	3.96	≥ 3.96
Margin MeanLin ratio	0.711 (0.609, 0.813)	0.408	1.43	≥ 1.43
Margin PE ratio	0.692 (0.586, 0.799)	0.342	1.55	≥ 1.55
Margin WiAUC ratio	0.700 (0.599, 0.801)	0.379	1.41	≥ 1.41
Margin WiPI ratio	0.696 (0.590, 0.801)	0.344	1.50	≥ 1.50
Margin WoAUC ratio	0.694 (0.596, 0.793)	0.381	1.32	≥ 1.32
Margin WiWoAUC ratio	0.699 (0.600, 0.798)	0.421	1.32	≥ 1.32
Variables	AUROC	Youden index	Cutoff value	Value tending to high Ki-67 pattern
Tumor MeanLin ratio	0.724 (0.631, 0.816)	0.428	2.98	≥ 2.98
Tumor PE ratio	0.690 (0.594, 0.786)	0.353	3.52	≥ 3.52
Tumor WiPI ratio	0.689 (0.593, 0.786)	0.335	6.52	≥ 6.52
Margin MeanLin ratio	0.750 (0.661, 0.839)	0.452	1.75	≥ 1.75
Margin PE ratio	0.759 (0.671, 0.848)	0.464	2.21	≥ 2.51
Margin WiAUC ratio	0.702 (0.608, 0.796)	0.379	1.54	≥ 1.54
Margin WiR ratio	0.741 (0.649, 0.833)	0.423	2.05	≥ 2.05
Margin WiPI ratio	0.758 (0.670, 0.847)	0.475	2.55	≥ 2.55
Margin WoR ratio	0.726 (0.634, 0.819)	0.367	2.29	≥ 2.55

Data in parentheses are 95% CIs.

CEUS, contrast-enhanced ultrasound; AUROC, area under the receiver operating characteristic; MTM, macrotrabecular-massive; MeanLin, average contrast signal intensity; PE, peak enhancement; WiAUC, wash-in area under the curve; WiR, wash-in rate; WiPI, wash-in perfusion index; WoAUC, wash-out AUC; WiWoAUC, wash-in and wash-out AUC; WoR, wash-out rate.

Table S4 Predictors of Clinic-CEUS models for MTM subtype and high Ki-67 expression pattern by logistic regression analysis in primary cohort.

Variables	Univariable OR	*p* value[a]	Multivariable OR	*p* value[b]
MTM-HCC subtype				
Serum AFP > 20 ng/mL	4.4 (1.9, 10.1)	< 0.001	4.5 (1.9, 10.9)	< 0.001
AFP-L3% > 10 U/mL	3.4 (1.5, 7.6)	0.004		0.215
Mean tumor size > 5 cm	2.1 (1.0, 4.7)	0.065		0.742
Intratumoral necrosis	2.8 (1.2, 6.2)	0.012	3.2 (1.3, 7.7)	0.010
Intratumoral artery	2.9 (1.2, 7.0)	0.017		0.847
Peritumoral nutrient vessel	2.5 (1.1, 5.6)	0.024	2.6 (1.1, 6.2)	0.035
AP heterogeneously enhancement	3.9 (1.3, 12.2)	0.019		0.124
AP branched enhancement	3.0 (1.1, 8.1)	0.034		0.877
PVP enhancement degree (Hypo-/Marked hypo-)	2.6 (1.1, 6.1)	0.025		0.214
High Ki-67 pattern				
Age < 59 years	3.0 (1.4, 6.4)	0.004	2.7 (1.2, 5.9)	0.013
Serum AFP > 20 ng/mL	2.3 (1.1, 5.0)	0.029		0.620
AFP-L3% > 10 U/mL	3.8 (1.7, 8.4)	0.001	3.4 (1.5, 7.8)	0.003
Intratumoral necrosis	2.3 (1.1, 5.0)	0.027		0.065
AP branched enhancement	4.3 (1.4, 12.9)	0.009	3.8 (1.2, 12.4)	0.028

Data in parentheses are 95% CIs.

[a] *p* values for univariable odds ratio.

[b] *p* values for Multivariable odds ratio.

MTM-HCC, macrotrabecular-massive hepatocellular carcinoma; OR, odds ratio; AFP, α-fetoprotein; AFP-L3%, lens culinaris agglutinin-bound fraction of α-fetoprotein; AP, arterial phase; PVP, portal vein phase.

Table S5 Logistic regression models for MTM subtype and high Ki-67 pattern.

Predictive model	Formula (presence = 1, absence = 0)	Cutoff
Clinic-CEUS model (MTM subtype)	1.512 × serum AFP > 20 ng/mL + 1.159 × intratumoral necrosis + 0.944 × peritumoral nutrient vessel - 2.453	0.25
Clinic-Q-CEUS model (MTM subtype)	1.425 × serum AFP > 20 ng/mL + 1.282 × PVP hypo-/marked hypo-enhancement + 1.957 × tumor MeanLin ratio ≥ 3.96 + 1.478 × margin MeanLin ratio ≥ 1.43 - 4.089	0.22
Clinic-CEUS model (High Ki-67 pattern)	0.993 × age < 59 years + 1.163 × serum AFP-L3% > 10 U/mL + 1.330 × AP branched enhancement pattern	0.32
Clinic-Q-CEUS model (High Ki-67 pattern)	1.128 × age < 59 years + 1.117 × serum AFP-L3% > 10 U/mL + 1.053 × tumor MeanLin ratio ≥ 2.98 + 1.563 × margin WiPI ratio ≥ 2.55 - 2.306	0.39

MTM, macrotrabecular-massive; Clinic-CEUS model, logistic regression model based on clinical data and contrast-enhanced ultrasound (CEUS) features; Clinic-Q-CEUS model, logistic regression model based on clinical data, CEUS features and dynamic CEUS quantitative parameters; AFP, α-fetoprotein; AFP-L3%, lens culinaris agglutinin-bound fraction of α-fetoprotein; PVP, portal vein phase; MeanLin, average contrast signal intensity; WiPI, wash-in perfusion index.
